# Hypoalbuminemia as a predictor of acute kidney injury during colistin treatment

**DOI:** 10.1038/s41598-018-30361-5

**Published:** 2018-08-10

**Authors:** Daniele Roberto Giacobbe, Alessandra di Masi, Loris Leboffe, Valerio Del Bono, Marianna Rossi, Dario Cappiello, Erika Coppo, Anna Marchese, Annarita Casulli, Alessio Signori, Andrea Novelli, Katja Perrone, Luigi Principe, Alessandra Bandera, Luca Enrico Vender, Andrea Misin, Pierpaolo Occhilupo, Marcello Melone, Paolo Ascenzi, Andrea Gori, Roberto Luzzati, Claudio Viscoli, Stefano Di Bella

**Affiliations:** 1 Infectious Diseases Unit, Ospedale Policlinico San Martino - IRCCS per l’Oncologia and Department of Health Science (DISSAL), University of Genoa, Genoa, Italy; 2 Department of Sciences, Section Biomedical Sciences and Technology, Roma Tre University, Rome, Italy; 3 Infectious Diseases Unit, Azienda Ospedaliera S. Croce e Carle, Cuneo, Italy; 4 Clinic of Infectious Diseases, San Gerardo Hospital, University of Milano-Bicocca, Monza, Italy; 5 Città di Lecce Hospital – GVM Care and Research, Lecce, Italy; 6 Microbiology Unit, University of Genoa (DISC) and Ospedale Policlinico San Martino - IRCCS per l’Oncologia, Genoa, Italy; 7 Department of Health Science (DISSAL), University of Genoa, Genoa, Italy; 8 Department of Health Sciences, Clinical Pharmacology and Oncology Section, University of Florence, Florence, Italy; 9 Clinical Microbiology and Virology Unit, A. Manzoni Hospital, Lecco, Italy; 10 Infectious Diseases Department, Azienda Sanitaria Universitaria Integrata di Trieste, Trieste, Italy; 11 Maria Cecilia Hospital – GVM Care & Research, Cotignola, Italy; 12 University of Milan and Infectious Diseases Unit, Department of Internal Medicine, Fondazione IRCCS Ca’ Granda Ospedale Maggiore Policlinico, Milan, Italy

## Abstract

This study aimed to assess the predictors of acute kidney injury (AKI) during colistin therapy in a cohort of patients with bloodstream infections (BSI) due to colistin-susceptible Gram-negative bacteria, focusing on the role of serum albumin levels. The study consisted of two parts: (1) a multicentre retrospective clinical study to assess the predictors of AKI during colistin therapy, defined according to the Kidney Disease: Improving Global Outcomes (KDIGO) criteria; and (2) bioinformatic and biochemical characterization of the possible interaction between human serum albumin and colistin. Among the 170 patients included in the study, 71 (42%), 35 (21%), and 11 (6%) developed KDIGO stage 1 (K1-AKI), KDIGO stage 2 (K2-AKI), and KDIGO stage 3 (K3-AKI), respectively. In multivariable analyses, serum albumin <2.5 g/dL was independently associated with K1-AKI (subdistribution hazard ratio [sHR] 1.85, 95% confidence interval [CI] 1.17–2.93, p = 0.009) and K2-AKI (sHR 2.37, 95% CI 1.15–4.87, p = 0.019). Bioinformatic and biochemical analyses provided additional information nurturing the discussion on how hypoalbuminemia favors development of AKI during colistin therapy. In conclusion, severe hypoalbuminemia independently predicted AKI during colistin therapy in a large cohort of patients with BSI due to colistin-susceptible Gram-negative bacteria. Further study is needed to clarify the underlying causal pathways.

## Introduction

Colistin is a polymyxin antibiotic with *in vitro* activity against most aerobic Gram-negative rods^1,2^. The use of colistin for the treatment of infections in humans was largely abandoned in the last quarter of the past century, owing to concerns of nephrotoxicity and neurotoxicity^1,3,4^. In the last two decades, the lack of dependable alternatives for treating infections due to multidrug resistant (MDR) Gram-negative bacteria has led to an increasing use of colistin, as well as to a renewed interest in its effectiveness and tolerability in humans^1,5,6^.

In recent years, several clinical studies have investigated risk factors for nephrotoxicity, or more appropriately acute kidney injury (AKI), during colistin treatment, with heterogeneous results. Indeed, possible increases in the risk of AKI due to increasing age, comorbid conditions, prolonged course of treatment, high colistin dosage, concomitant nephrotoxic agents, pre-existing renal failure, hypertension, and obesity have been variously reported^6–29^. In addition, hypoalbuminemia has also been suggested to possibly predispose to AKI during colistin therapy^15,18,24,27^. However, the true impact of hypoalbuminemia remains unclear because of conflicting results in different studies, possibly relying on the wide heterogeneity in patient population, type of infections, and study design.

In the present study, we assessed the predictors of AKI during colistin therapy in a cohort of patients with bloodstream infections (BSI) due to colistin-susceptible Gram-negative bacteria, focusing on the role of serum albumin levels. In addition, we performed docking simulations and biochemical characterization of colistin binding to human serum albumin (HSA), in order to verify whether an interaction between colistin and albumin exist that might add to the discussion on any possible causal relationship between hypoalbuminemia and AKI during colistin therapy.

## Materials and Methods

The present study consists of two parts: (1) a multicentre retrospective clinical study; and (2) bioinformatic and biochemical characterization of the possible interaction between human serum albumin (HSA) and colistin.

### Part 1 – Multicentre retrospective clinical study

#### Study design

The multicentre retrospective clinical study was conducted in the following Italian hospitals: (*i*) Ospedale Policlinico San Martino – IRCCS per l’Oncologia, 1200 beds, in Genoa; (*ii*) Azienda Sanitaria Universitaria Integrata di Trieste, 600 beds, in Trieste; (*iii*) ASST Monza – Ospedale San Gerardo, 1200 beds, in Monza; and (*iv*) Città di Lecce Hospital – gruppo GVM Care and Research, 150 beds, in Lecce. All patients with BSI caused by colistin-susceptible Gram-negative bacteria and treated with intravenous colistin from January 2011 to June 2016 were identified through the computerized databases of the four hospitals. Exclusion criteria were: (*i*) missing key data; (*ii*) less than 48 h of intravenous colistin; and (*iii*) hemodialysis. The primary study outcome measure was AKI during colistin treatment, defined as a time-to-event endpoint. Patients were followed until the 14^th^ day of colistin therapy or death, whichever came first.

#### Definitions

BSI was defined as the presence of at least one blood culture positive for Gram-negative bacteria in presence of signs and symptoms of infection^30^. AKI was defined on the basis of serum creatinine levels according to the Kidney Disease: Improving Global Outcomes (KDIGO) criteria (Table 1)^31^.

**Table 1:** KDIGO stages of acute kidney injury according to serum creatinine levels.

Stage	Description
Stage 1	Increase in serum creatinine by ≥0.3 mg/dl within 48 hours or increase in serum creatinine 1.5 to 1.9 times baseline which is known or presumed to have occurred within the prior 7 days
Stage 2	Increase in serum creatinine to 2.0 to 2.9 times baseline
Stage 3	Increase in serum creatinine to 3.0 times baseline or increase in serum creatinine to ≥4.0 mg/dl or initiation of renal replacement therapy

KDIGO, Kidney Disease: Improving Global Outcomes^31^.

#### Data collection

The following demographic and clinical variables were collected from clinical charts as baseline data at the time of colistin initiation: age; gender; presence of diabetes mellitus, presence of solid neoplasms, presence of hematological malignancies, presence of chronic renal failure, presence of severe hepatic failure (defined as liver cirrhosis according to histology or in presence of a clinical diagnosis supported by laboratory, endoscopy, and radiologic findings^32^), presence of septic shock (defined as hypotension not responding to fluid therapy and requiring vasoactive agents), presence of polymicrobial BSI, ward of stay (intensive care unit [ICU] vs. non-ICU), presence of central venous catheter, neutropenia (defined as absolute neutrophil count of <500/mm^3^), serum total bilirubin, serum hemoglobin, serum creatinine, serum albumin. The following data were also collected with regard to the management and course of BSI: dosage and length of colistin therapy, whether colistin was given as monotherapy or in combination, time to adequate therapy (defined as the number of days elapsing from the first positive blood culture to initiation of at least one antibiotic with *in vitro* activity against the causative agent of BSI), serum creatinine during colistin therapy.

#### Microbiology

The Vitek 2 automated system (bioMérieux, Marcy l’Etoile, France) was routinely used for identifying the causative agent of BSI and for susceptibility testing. Colistin susceptibility testing was performed using the Vitek 2 system or the Etest (bioMérieux, Marcy l’Etoile, France). Susceptibility results were interpreted according to the latest EUCAST criteria (EUCAST breakpoint tables for interpretation of MICs and zone diameters, version 6.0, 2016; http://www.eucast.org).

#### Statistical analysis

Baseline demographic and clinical data of patients were described with numbers and percentages for categorical variables, and with median and interquartile range (IQR) for continuous variables.

The main analysis was the identification of predictors of AKI during colistin therapy. To this aim, the possible association of demographic and clinical factors with AKI was assessed in univariable Fine-Gray models, with AKI as the outcome of interest and death as a competing event^33^. The Day 0 was the day of colistin initiation. Patients who discontinued colistin before 14 days of therapy were right-censored at the time of discontinuation. In different univariable models, serum albumin was either dichotomized according to an arbitrary cut-off based on its median concentration in the study population (<2.5 g/dl vs. ≥2.5 g/dl), or considered as a continuous variable. All the variables were tested for their possible association with three different dependent variables, reflecting the three different stages of AKI according to KDIGO classification: (*i*) KDIGO stage 1 (K1-AKI); (*ii*) KDIGO stage 2 (K2-AKI); (*iii*) KDIGO stage 3 (K3-AKI).

To assess the independent role of variables, factors showing a potential association with AKI in univariable analyses (p < 0.10) were included in an initial multivariable Fine-Gray model, and further selected through a stepwise backward procedure based on the Akaike information criterion. Different multivariable analyses were conducted according to all the possible combinations of independent (dichotomous or continuous albumin) and dependent (K1-AKI, K2-AKI, K3-AKI) variables. The proportionality assumption was checked by plotting Schoenfeld residuals and by verifying the absence of statistically significant interactions between time and covariates in Fine-Gray models.

The cumulative incidence of K1-AKI, K2-AKI, and K3-AKI, in patients with or without severe hypoalbuminemia (<2.5 and ≥2.5 g/dL, respectively) was calculated by means of the Aalen-Johansen method, considering death as a competing event and applying right-censoring at the time of colistin discontinuation^34^. Statistical analyses were performed using the R Statistical Software (version 3.3.0, R Foundation for Statistical Computing, Vienna, Austria).

### Part 2 - Bioinformatic and biochemical analyses

#### Bioinformatic analysis

Docking simulations of colistin binding to HSA were performed using the crystal structure of ligand-free HSA (PDB ID: 1AO6)^35^. The colistin three-dimensional structure was obtained from the Drug Bank Database (https://www.drugbank.ca/drugs/DB00803). Simulations were carried out using DockingApp^36^, a user friendly interface for the docking program AutoDock Vina^37^. In all the simulations, the search space (docking grid) included the whole HSA structure in order to carry out “blind” predictions of the colistin binding sites. The simulations were carried out both by keeping all protein residues rigid and by allowing flexibility only of the residues building up the walls of the FA sites (FA1 to FA9) (see^38^). Residues for which flexibility was allowed are reported in the supplementary material (Table S1). Rotatable bonds of colistin structure were kept flexible in all the simulations.

#### Biochemical analysis

As spectrofluorimetric and spectrophotometric binding studies were not informative of colistin binding to HSA due to the lack of optical variations following the interaction, we performed an indirect assay. In particular, colistin binding to HSA was evaluated by measuring the MG1655 *E. coli* strain (ATCC® 47046; Manassas, VA, USA) growth. Briefly, the activity of colistin (Sigma-Aldrich, St. Louis, MO, USA) and HSA (Sigma-Aldrich) on the MG1655 *E. coli* strain was tested in 96-well microtiter plates. In order to obtain high cell densities, bacterial cells were grown overnight in Mueller Hinton Broth 2 (MH II) (Sigma-Aldrich) and then diluted to an OD600 of 0.001 in MH II broth containing increasing concentrations of colistin (1, 1.25, and 1.5 μg/mL) and HSA (25, 50, 100, and 200 μg/mL). Microtiter plates were incubated for 20 h at 37 °C. Bacteria growth was measured at a wavelength of 600 nm using a microplate reader (Spark, Tecan, Switzerland). Biochemical results are shown as the means ± standard deviation (SD) derived minimally from three independent experiments. Differences between means, assessed by the Student’s t-test (GraphPad InStat 3.1 Software Inc., San Diego, CA, USA), were considered significant when p values were ≤0.05.

### Ethical approval and informed consent

All procedures performed were in accordance with the ethical standards of the institutional and/or national research committees and with the 1964 Helsinki Declaration and its later amendments or comparable ethical standards. The study was approved by the regional ethics committee of the coordinating center (Regional Ethics Committee of Liguria Region, registry number 473REG2016) and subsequently by the local ethics committees/institutional review boards of the other participating centers. No specific informed consent was required because of the retrospective nature of the analyses.

### Availability of data and materials

The datasets used and/or analyzed during the current study are available from the corresponding author on reasonable request.

## Results

### Part 1 – Multicentre retrospective clinical study

**Figure 1:**
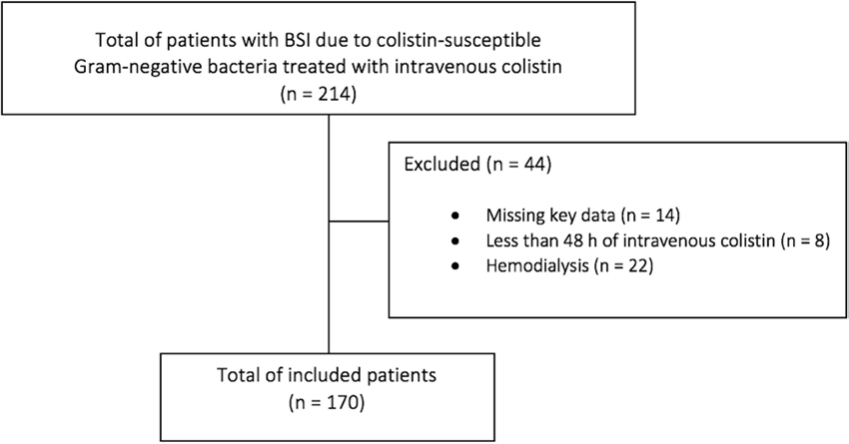
Flow chart of the patients inclusion process.

During the study period, 214 patients with BSI due to colistin-susceptible Gram-negative bacteria were treated with intravenous colistin, and 170 of them (79%) were included in the study (Fig. 1). Their median age was 68 years (IQR 58–76), and 111 were males (65%). Their complete demographic and clinical characteristics are reported in Table 2. Overall, 71 (42%), 35 (21%), and 11 (6%) patients developed K1-AKI, K2-AKI, and K3-AKI during colistin therapy, respectively. Crude mortality within 14 days from the first positive blood culture was 17% (29/170).

**Table 2:** Baseline characteristics of the study population.

Variable	Patients not developing AKI during colistin treatment (%) 99 (58)	Patients developing at least KDIGO Stage 1 AKI during colistin treatment (%) 71 (42)
**Demographic variables**		
Age in years, median (IQR)	67 (58–75)	71 (60–77)
Male gender	67 (68)	44 (62)
**Medical hystory**		
Diabetes mellitus	25 (25)	23 (32)
Chronic renal failure	13 (13)	15 (21)
Severe hepatic failure	11 (11)	3 (4)
Solid neoplasms	25 (25)	11 (15)
Hematological malignancies	17 (17)	9 (13)
**Microbiological variables**		
Causative agent of BSI		
Enterobacteriaceae^§^	65 (66)	37 (52)
*Pseudomonas* spp.	13 (13)	11 (15)
*Acinetobacter* spp.	15 (15)	18 (25)
Enterobacteriaceae plus *Pseudomonas* spp.^§§^	3 (3)	4 (6)
Enterobacteriaceae plus *Acinetobacter* spp.^§§§^	3 (3)	1 (1)
Polymicrobial BSI*	23 (23)	14 (20)
Colistin susceptibility test method		
Vitek 2	72 (73)	51 (72)
Etest	27 (27)	20 (28)
Baseline variables**		
ICU stay	27 (27)	19 (27)
Presence of central venous catheter	83 (84)	54 (76)
Presence of septic shock	34 (34)	24 (34)
Neutropenia	14 (14)	3 (4)
Serum hemoglobin in g/dl, median (IQR)	9.3 (8.7–10.1)	9.3 (8.6–10.3)
Serum total bilirubin in mg/dl, median (IQR)	0.8 (0.5–1.3)	0.9 (0.5–1.6)
Serum creatinine in mg/dl, median (IQR)	0.9 (0.6–1.4)	1.0 (0.7–1.4)
Serum albumin in g/dl, median (IQR)	2.6 (2.3–3.0)	2.4 (2.1–2.8)
Serum albumin <2.5 g/dl	38 (38)	42 (59)
**Therapy variables**		
Type of adequate therapy		
Colistin monotherapy	51 (52)	36 (51)
2 active agents including colistin	42 (42)	31 (44)
3 active agents including colistin	6 (6)	4 (6)
Time to adequate therapy in days, median (IQR)	2 (0–4)	3 (1–4)
9 MU colistimethate loading-dose***	54 (55)	35 (49)
Length of colistin therapy in days, median (IQR)	13 (10–20)	13 (8–15)
Use of other nephrotoxic agents****	7 (7)	6 (8)

Results are presented as n (%) unless otherwise indicated. KDIGO, Kidney Disease: Improving Global Outcomes^31^; AKI, acute kidney injury; IQR, Interquartile range; ICU, intensive care unit; MU, million units.

^§^*Klebsiella* spp. (n = 95), *Citrobacter* spp. (n = 1), *Enterobacter* (n = 1), *Enterobacter* spp. plus *Klebsiella* spp. (n = 1), *Escherichia* spp. (n = 1), *Klebsiella* spp. plus *Proteus* spp. (n = 3).

^§§^*Klebsiella* spp. plus *Pseudomonas* spp. (n = 6), *Proteus* spp. plus *Pseudomonas* spp. (n = 1).

^§§§^ *Acinetobacter* spp. plus *Citrobacter* spp. (n = 1), *Acinetobacter* spp. plus *Escherichia* spp. (n = 1), *Acinetobacter* spp. plus *Escherichia* spp. plus *Klebsiella* spp. (n = 1), *Acinetobacter* spp. plus *Proteus* spp. (n = 1).

*Acinetobacter spp. plus *Enterococcus* spp. (n = 7), *Klebsiella* spp. plus *Enterococcus* spp. (n = 5), *Klebsiella spp*. plus *Pseudomonas spp*. (n = 5), *Klebsiella* spp. plus *Proteus* spp. (n = 3), *Pseudomonas* spp. plus *Enterococcus* spp. (n = 2), *Acinetobacter* spp. plus *Candida* spp. (n = 1), *Acinetobacter* spp. plus *Citrobacter* spp. (n = 1), *Acinetobacter* spp. plus coagulase-negative staphylococci (n = 2), *Acinetobacter* spp. plus *Escherichi*a spp. (n = 1), *Acinetobacter* spp. plus *Escherichia* spp. plus *Klebsiella* spp. plus *Enterococcus* spp. (n = 1), *Acinetobacter* spp. plus *Proteus* spp. (n = 1), *Acinetobacter* spp. plus *Staphylococcus aureus* (n = 1), *Citrobacter* spp. plus coagulase-negative staphylococci (n = 1), *Enterobacter* spp. plus *Klebsiella* spp. (n = 1), *Klebsiella* spp. plus *Candida* spp. (n = 1), *Klebsiella* spp. plus coagulase-negative staphylococci (n = 1), *Proteus* spp. plus *Pseudomonas* spp. (n = 1), *Pseudomonas* spp. plus coagulase-negative staphylococci (n = 1), *Pseudomonas* spp. plus *Klebsiella* spp. plus *Enterococcus* spp. (n = 1).

**At the time of colistin initiation.

***Colistimethate maintenance dose was mostly administered at 4.5 MU twice daily (99/170, 58%) and 3 MU thrice daily (9/170, 5%); various dosages with reduced amount/frequency of administration of colistimethate were used for patients with increased serum creatinine levels.

****Gentamicin (n = 11), amikacin (n = 1), vancomycin (n = 1).

Table 3 shows univariable and multivariable analysis of predictors of K1-AKI during colistin therapy, considering baseline serum albumin as a dichotomous variable (<2.5 g/dL vs. ≥2.5 g/dL). In univariable comparisons, increasing age and serum albumin <2.5 g/dL were associated with K1-AKI. In multivariable analysis, only serum albumin <2.5 g/dL remained independently associated with K1-AKI (subdistribution hazard ratio [sHR] 1.85, 95% confidence interval [CI] 1.17–2.93, p = 0.009). As a continuous variable, serum albumin showed an association with K1-AKI in univariable analysis (sHR 0.63 for each increase of 1 g/dL in baseline serum albumin, 95% IC 0.42–0.93, p = 0.021), whereas statistical significance was not retained in the final multivariable model (sHR 0.68 for each increase of 1 g/dL in baseline serum albumin, 95% IC 0.45–1.03, p = 0.066; for details see Supplementary Table S2).

**Table 3:** Univariable and multivariable analyses of factors associated with development of acute kidney injury (KDIGO stage 1).

Variable	Univariable analysis		Multivariable analysis*	
	sHR (95% CI)	p	sHR (95% CI)	p
Age in years	1.02 (0.98–1.04)	0.046		
Male gender	0.86 (0.54–1.36)	0.51		
Diabetes mellitus	1.35 (0.84–2.17)	0.22		
Chronic renal failure	1.64 (0.93–2.9)	0.09		
Severe hepatic failure	0.40 (0.13–1.25)	0.11		
Solid neoplasms	0.64 (0.34–1.21)	0.17		
Hematological malignancies	0.75 (0.38–1.49)	0.42		
Causative agent of BSI		0.26		
Enterobacteriaceae	1 (Reference)			
*Pseudomonas* spp.	1.31 (0.69–2.50)			
*Acinetobacter* spp.	1.78 (1.03–3.07)			
Enterobacteriaceae plus *Pseudomonas* spp.	1.79 (0.66–4.83)			
Enterobacteriaceae plus *Acinetobacter* spp.	0.74 (0.12–4.53)			
Polymicrobial BSI	0.84 (0.48–1.46)	0.53		
Colistin susceptibility test method		0.85		
Vitek 2	1 (Reference)			
Etest	1.05 (0.63–1.73)			
ICU stay	1.00 (0.60–1.68)	0.99		
Presence of central venous catheter	0.70 (0.41–1.19)	0.18		
Presence of septic shock	1.07 (0.66–1.73)	0.79		
Neutropenia	0.33 (0.11–1.02)	0.054	0.40 (0.13–1.26)	0.12
Serum hemoglobin in g/dl	1.05 (0.86–1.29)	0.62		
Serum total bilirubin in mg/dl	1.00 (0.95–1.05)	0.88		
Serum creatinine in mg/dl	1.17 (0.96–1.42)	0.13		
Serum albumin<2.5 g/dl	1.99 (1.26–3.14)	0.003	1.85 (1.17–2.93)	0.009
Type of adequate therapy		0.93		
Colistin monotherapy	1 (Reference)			
2 active agents including colistin	1.04 (0.65–1.66)			
3 active agents including colistin	0.87 (0.36–2.14)			
Time to adequate therapy in days	1.03 (0.95–1.12)	0.44		
9 MU colistimethate loading-dose	0.88 (0.56–1.38)	0.58		
Use of other nephrotoxic agents	1.08 (0.51–2.31)	0.84		

KDIGO, Kidney Disease: Improving Global Outcomes^31^; sHR, subdistribution hazard ratio; CI, confidence intervals; BSI, bloodstream infection; ICU, intensive care unit; MU, million units.

*Only results for variables retained in the final multivariable model are presented.

As shown in Table 4, serum albumin <2.5 g/dL resulted also the only independent predictor of K2-AKI (sHR 2.37, 95% CI 1.15–4.87, p = 0.019). As a continuous variable, serum albumin was associated with K2-AKI both in univariable (sHR 0.52 for each increase of 1 g/dL in baseline serum albumin, 95% IC 0.31–0.88, p = 0.014) and in multivariable (sHR 0.57 for each increase of 1 g/dL in baseline serum albumin, 95% IC 0.34–0.95, p = 0.03) models, as detailed in Supplementary Table S3.

**Table 4:** Univariable and multivariable analyses of factors associated with development of acute kidney injury (KDIGO stage 2).

Variable	Univariable analysis		Multivariable analysis*	
	sHR (95% CI)	p	sHR (95% CI)	p
Age in years	1.03 (1.00–1.06)	0.092	1.02 (0.99–1.06)	0.17
Male gender	0.95 (0.49–1.87)	0.89		
Diabetes mellitus	1.18 (0.59–2.36)	0.64		
Chronic renal failure	0.79 (0.32–1.99)	0.62		
Severe hepatic failure	0.70 (0.17–2.97)	0.63		
Solid neoplasms	1.28 (0.58–2.82)	0.54		
Hematological malignancies	0.71 (0.26–1.91)	0.49		
Causative agent of BSI		0.67		
Enterobacteriaceae	1 (Reference)			
*Pseudomonas* spp.	1.15 (0.43–3.05)			
*Acinetobacter* spp.	1.72 (0.82–3.62)			
Enterobacteriaceae plus *Pseudomonas* spp.	1.80 (0.37–8.91)			
Enterobacteriaceae plus *Acinetobacter* spp.	1.66 (0.27–10.36)			
Colistin susceptibility test method		0.13		
Vitek 2	1 (Reference)			
Etest	1.69 (0.85–3.35)			
Polymicrobial	0.55 (0.21–1.43)	0.22		
ICU stay	1.63 (0.83–3.2)	0.16		
Presence of central venous catheter	0.47 (0.23–0.95)	0.035	0.56 (0.26–1.23)	0.15
Presence of septic shock	1.17 (0.59–2.29)	0.66		
Neutropenia	0.28 (0.04–1.93)	0.2		
Serum hemoglobin in g/dl	1.05 (0.83–1.35)	0.67		
Serum total bilirubin in mg/dl	0.97 (0.90–1.05)	0.48		
Serum creatinine in mg/dl	0.73 (0.38–1.42)	0.35		
Serum albumin < 2.5 g/dl	2.55 (1.27–5.15)	0.009	2.37 (1.15–4.87)	0.019
Type of adequate therapy		0.69		
Colistin monotherapy	1 (Reference)			
2 active agents including colistin	0.75 (0.38–1.51)			
3 active agents including colistin	0.90 (0.26–4.72)			
Time to adequate therapy in days	1.02 (0.91–1.15)	0.7		
9 MU colistimethate loading-dose	1.82 (0.92–3.6)	0.086	1.83 (0.88–3.82)	0.11
Use of other nephrotoxic agents	0.87 (0.20–3.77)	0.85		

KDIGO, Kidney Disease: Improving Global Outcomes^31^; sHR, subdistribution hazard ratio; CI, confidence intervals; BSI, bloodstream infection; ICU, intensive care unit; MU, million units.

*Only results for variables retained in the final multivariable model are presented.

With regard to the potential association of serum albumin with K3-AKI, despite a trend towards increased risk of K3-AKI in patients with serum albumin <2.5 g/dL, no statistically significant association was detected in univariable analysis (sHR 3.16, 95% CI 0.85–11.8, p = 0.086). A similar result was observed when serum albumin was considered as a continuous variable (sHR 0.46 for each increase of 1 g/dL in baseline serum albumin, 95% CI 0.20–1.05, p = 0.064). The details of univariable analyses for K3-AKI are available in Supplementary Table S4. No multivariable analysis was conducted because of the low number of K3-AKI events.

**Figure 2:**
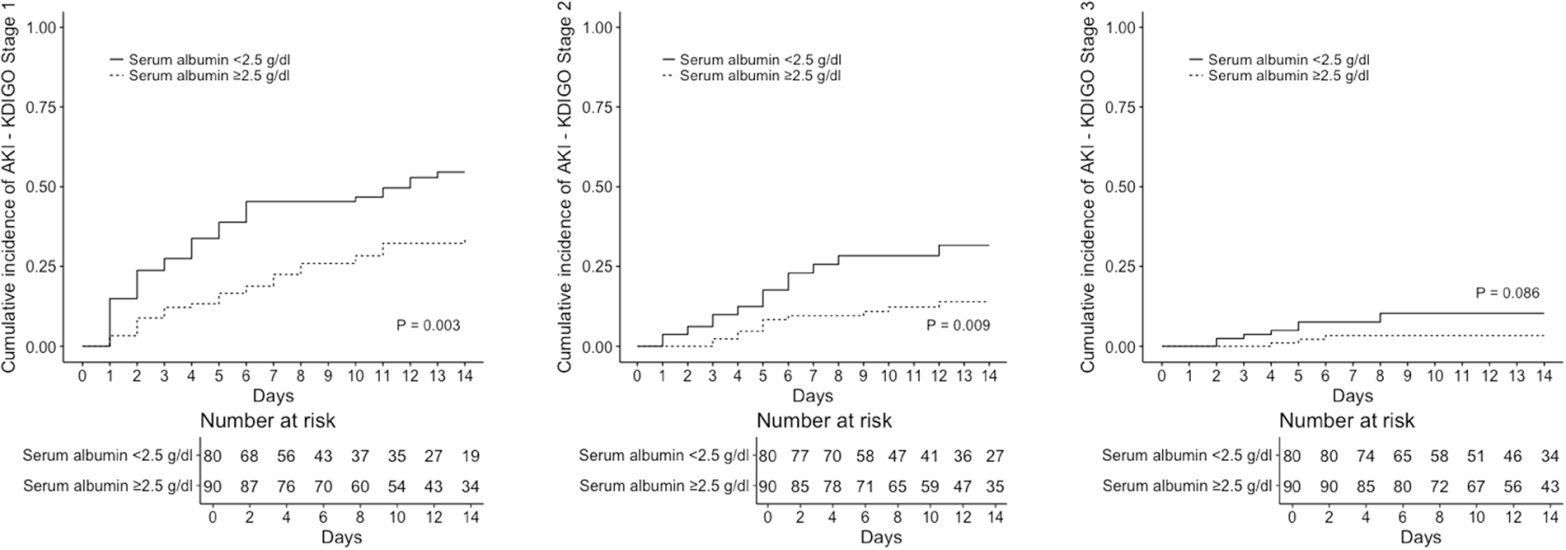
Cumulative incidence of acute kidney injury in the study population. KDIGO, Kidney Disease: Improving Global Outcomes^31^; AKI, acute kidney injury.

The cumulative incidence of K1-AKI, K2-AKI, and K3-AKI in patients with serum albumin <2.5 g/dL and ≥2.5 g/dL is shown in Fig. 2.

### Part 2 - Bioinformatic and biochemical analyses

**Figure 3:**
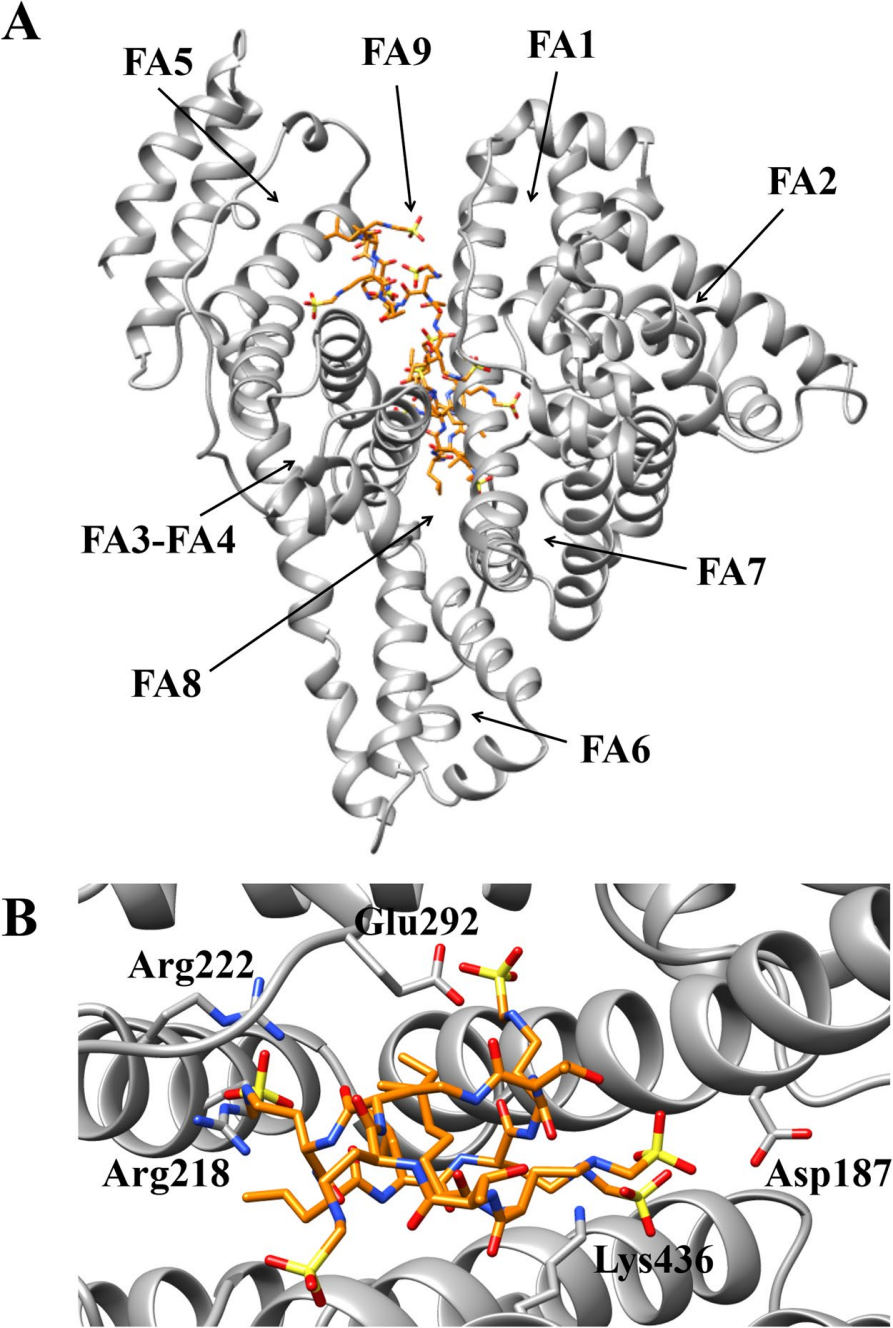
Colistin recognition by ligand-free HSA. (**A**) Overall view of the best apparent free energy poses of ligand-free HSA-colistin complexes. (**B**) Overall view of the atomic details of colistin recognition at the FA8 site of HSA. Colistin is rendered in stick (orange). The picture was drawn with the UCSF-Chimera package^39^. For details, see text.

Docking simulations of colistin binding to ligand-free HSA, with the search space extended to the whole protein, indicated the preferential binding of this drug to the FA8 site, with a number of five complexes observed in a maximum of nine poses (Fig. 3A and Table 5)^39^. In particular, colistin recognition to the FA8 site of ligand-free HSA is based on hydrogen bonds with Asp187, Arg218, Arg222, Glu292, and Lys436 (Fig. 3B). Of note, this binding mechanism is reminiscent of that observed for thyroxine recognition^40^. In other poses of docking simulation, colistin has been found to be placed in the FA9 site, which is located in the upper region of the FA8 site (Fig. 3A and Table 5)^41,42^.

**Table 5:** Docking simulation of colistin binding to ligand-free HSA.

Pose	Ligand-free HSA (PDB ID: 1AO6)		Ligand-free HSA flex (PDB ID: 1AO6)	
	Affinity (kcal/mol)	Site	Affinity (kcal/mol)	Site
1	−6.7	FA8	−7.9	FA8
2	−6.6	FA8	−7.8	FA8
3	−6.6	FA9	−7.4	FA8
4	−6.6	FA9	−7.4	FA8
5	−6.6	FA8	−7.4	FA9
6	−6.5	FA9	−7.3	FA8
7	−6.5	FA8	−7.3	FA9
8	−6.5	FA8	−7.3	FA9
9	−6.5	FA9	−7.3	FA8

As shown in Fig. S1, *E. coli* growth was completely inhibited in the presence of colistin, at all the concentrations tested. The addition of HSA to the broth significantly reduced the anti-microbial effect of colistin as a consequence of the reduced bioavailability due to its binding to albumin. Of note, the addition of HSA to the bacteria broth did not affect *E. coli* growth (Fig. S1).

## Discussion

In a retrospective cohort of 170 patients with BSI due colistin-susceptible Gram-negative bacteria treated with colistin, as many as 42% developed at least mild AKI during colistin therapy, and 6% developed severe AKI. Severe hypoalbuminemia at colistin initiation (<2.5 g/dL) was associated with the development of AKI during colistin treatment.

Although with the necessary premise that direct comparisons are somewhat hampered by the use of different definitions of AKI in different studies, it is of note that the rates of AKI during colistin therapy detected in our cohort seem coherent with current literature^43,44^. For example, in a very recent study in 149 critically-ill patients receiving intravenous colistin, decreases of ≥25%, ≥50% and ≥75% in creatinine clearance during colistin treatment were observed in 49%, 39%, and 8% of cases, respectively^44^. These results likely reflect the low therapeutic index of colistin^45^, and in turn the need for proper use and prompt identification of factors favoring the development of AKI during the course of treatment.

Other authors have already investigated hypoalbuminemia as a possible predictor of AKI during colistin therapy. In two previous studies, serum albumin concentrations <3.2 g/dL and <2 g/dL were associated with development of AKI during treatment in 47 and 71 patients with various types of infections, respectively^18,27^. Low serum albumin concentrations also predicted AKI during colistin therapy in other 42 patients with heterogeneous infections^15^. In the study of Lee and colleagues, lower serum albumin concentrations were associated with the development in AKI in 285 colistin-treated patients with estimated glomerular filtration rate (GFR) ≥ 60 mL/min/1.73 m^2^, but not in 44 patients with estimated GFR < 60 mL/min/1.73 m^2^ (although possibly because of the low sample size of this latter subgroup)^24^. Finally, Sorlì and colleagues observed that in 102 colistin-treated patients with various types of infections, hypoalbuminemia was associated with the development of AKI in univariable but not in multivariable analyses^17^. These heterogeneous results might rely, at least in part, on the different patient populations analyzed in the different studies, including various combinations of critically- and non-critically-ill patients, as well as various types of infections and causative agents. In the present study, we observed an independent association between severe hypoalbuminemia (<2.5 g/dL) and development of AKI during colistin therapy in an cohort of 170 patients with BSI due to colistin-susceptible Gram-negative bacteria. Notably, these results suggest that severe hypoalbuminemia is a true risk factor for AKI in those patients in whom colistin is probably used the most nowadays, and in whom it may remain the only active therapeutic option^46–48^.

The binding of colistin to albumin has also been evaluated in the present work, thereby providing some insights into their interaction in the serum of colistin-treated patients. Our bioinformatics and biochemical analyses demonstrated that colistin binds to albumin with a very high affinity, as also supported by the *in vitro E. coli* growth assay. However, as far as a causal relationship is concerned, it is important to note that this does not automatically imply an increased colistin nephrotoxicity because of reduced albumin binding in the serum of hypoalbuminemic patients. Indeed, as already observed by Nation and colleagues, hypoalbuminemia might promote reductions in the total concentration of colistin in serum (bound plus unbound) but perhaps not in that of the unbound drug, which is ultimately the toxic entity^49^. In this light, other possible explanations for the increased risk of AKI in hypoalbuminemic patients receiving colistin therapy should be necessarily considered. For example, severe hypoalbuminemia is responsible for altered regulation of fluids (and drugs) distribution, which might jeopardize renal perfusion and colistin ability to clear the infection and prevent/reduce any infection-related kidney injury^50^. In addition, hypoalbuminemia might also contribute to AKI through reduced anti-oxidant activities, less efficacious scavenging of reactive oxygen species, and impaired preservation of renal tubular cells^51,52^. Finally, hypoalbuminemia might simply be a proxy for poor clinical conditions, thus potentially influencing the development of AKI independent of any binding with colistin. With so many different but reasonable explanations at stake, it is therefore plausible that the development of AKI in hypoalbuminemic patients during colistin therapy is a complex and multifactorial process, in which not only the magnitude but also the existence of any possible contribution of reduced albumin binding still remains unclear. However, we think our findings nonetheless add to the literature, nurturing the discussion on the possible mechanisms trough which hypoalbuminemia leads to AKI in colistin-treated patients, and providing baseline biochemical information that might help in designing further dedicated studies to enrich our knowledge.

This study has some important limitations. The major one is that we did not have retrospective data on serum colistin levels, a well-known factor that may independently influence the development of AKI^45^. This also prevented us from assessing whether or not severe hypoalbuminemia influenced colistin concentrations in a clinically significant way^49,53^. Another potential confounding factor we could not adequately explore retrospectively is intravenous albumin therapy, which might have altered serum albumin levels (and their impact on AKI) in a time-dependent manner during colistin treatment. However, it is worth noting that intravenous albumin was likely administered the most in the case of severe hypoalbuminemia, which remained associated with AKI despite any possible interfering effect of subsequent corrections of serum albumin levels. This indirectly testifies to the absence of important biases related to albumin administration. It should also be noted that broth microdilution is the EUCAST reference method for colistin susceptibility testing, and not the Etest or the Vitek 2 automated system. However, although some patients with resistant strains might have been included because of the use of the Etest^54^, it should be noted that the majority of patients were selected according to Vitek 2 results. Reasonably, this did not hinder their correct selection, since colistin MICs are usually overestimated rather than underestimated by the Vitek 2 system^55^. Finally, we decided to use Fine-Gray regression with death as a competing event since our study was focused on the impact of hypoalbuminemia on the development of AKI. Indeed, hypoalbuminemia had been previously associated with increased mortality^28,56,57^, and we were thus interested not only in whether hypoalbuminemia predisposes to AKI, but also in whether it predisposes first to AKI than to mortality. However, the interpretation of the possible impact on AKI of other variables, which were not the primary focus of our study, might be less immediate than usual when using competing risk methods^58^. Therefore, any related conclusion should be drawn cautiously from this study.

## Conclusions

Severe hypoalbuminemia was an independent predictor of AKI during colistin therapy in a large cohort of patients with BSI due to colistin-susceptible Gram-negative bacteria. Further study is needed to clarify the underlying causal pathways.

## Electronic supplementary material

Supplementary material
