# Supplementary material for: Hypoalbuminemia as a predictor of acute kidney injury during colistin treatment
Source: Sci Rep. 2018 Aug 10;8:11968. doi: 10.1038/s41598-018-30361-5 (PMC6086859; doi:10.1038/s41598-018-30361-5)
Supplement: Supplementary file 1 — Supplementary material [file 41598_2018_30361_MOESM1_ESM.pdf]

Hypoalbuminemia as a predictor of acute kidney injury during colistin treatment

Daniele Roberto Giacobbe*[1], Alessandra di Masi[2], Loris Leboffe[2], Valerio Del Bono[3], Marianna Rossi[4], Dario Cappiello[5], Erika Coppo[6], Anna Marchese[6], Annarita Casulli[1], Alessio Signori[7], Andrea Novelli[8], Katja Perrone[5], Luigi Principe[9], Alessandra Bandera[4], Luca Enrico Vender[4], Andrea Misin[10], Pierpaolo Occhilupo[11], Marcello Melone[5], Paolo Ascenzi[2], Andrea Gori[12], Roberto Luzzati[10], Claudio Viscoli[1], Stefano Di Bella[10]

[1] Infectious Diseases Unit, Ospedale Policlinico San Martino - IRCCS per l'Oncologia and Department Health Science (DISSAL), University of Genoa, Genoa, Italy

[2] Department of Sciences, Section Biomedical Sciences and Technology, Roma Tre University, Rome, Italy

[3] Infectious Diseases Unit, Azienda Ospedaliera S. Croce e Carle, Cuneo, Italy.

[4] Clinic of Infectious Diseases, San Gerardo Hospital, University of Milano-Bicocca, Monza, Italy

[5] Città di Lecce Hospital – GVM Care and Research, Lecce, Italy

[6] Microbiology Unit, University of Genoa (DISC) and Ospedale Policlinico San Martino - IRCCS per l'Oncologia, Genoa, Italy

[7] Department Health Science (DISSAL), University of Genoa, Genoa, Italy

[8] Department of Health Sciences, Clinical Pharmacology and Oncology Section, University of Florence, Florence, Italy

[9] Clinical Microbiology and Virology Unit, A. Manzoni Hospital, Lecco, Italy

[10] Infectious Diseases Department, Azienda Sanitaria Universitaria Integrata di Trieste, Trieste, Italy

[11] Maria Cecilia Hospital – GVM Care & Research, Cotignola, Italy

[12] University of Milan and Infectious Diseases Unit, Department of Internal Medicine, Fondazione IRCCS Ca' Granda Ospedale Maggiore Policlinico, Milan, Italy

* Address correspondence to:

Daniele Roberto Giacobbe, MD

Ospedale Policlinico San Martino - IRCCS per l'Oncologia

Department Health Science (DISSAL), University of Genoa

L.go R. Benzi, 10 – 16132 Genoa, Italy

Telephone: +39 010 555 4654; Fax: +39 010 5556606

Email address: daniele.roberto.giacobbe@gmail.com

Table S1. Residues for which flexibility was allowed in docking simulations of colistin binding to the fatty acid binding sites of HSA

FA binding site	Residues
FA1	Arg117, Leu182, Tyr161
FA2	Tyr150, Arg257, Ser287, Ala254
FA3	Ser342, Arg485, Arg348, Leu453
FA4	Tyr411, Ser489, Arg410, Leu457, Phe488, Val415, Leu460
FA5	Tyr 401, Lys525, Met548, Phe551
FA6	Arg209, Lys351, Ser480, Asp324, Glu354, Ala213
FA7	Lys199, Arg218, Arg222, His242, Val241
FA8	Lys195, Asp451, Ser454
FA9	Asp187, Lys432

Table S2. Multivariable analysis of factors associated with development of acute kidney injury (KDIGO stage 1) considering serum albumin as a continuous variable

Variable	sHR (95% CI)	P
Neutropenia	0.39 (0.12-1.24)	0.11
Serum albumin in g/dl	0.68 (0.45-1.03)	0.066

KDIGO, Kidney Disease: Improving Global Outcomes [1]; sHR, subdistribution hazard ratio; CI, confidence intervals.

Only results for variables retained in the final multivariable model are presented

Table S3. Multivariable analysis of factors associated with development of acute kidney injury (KDIGO stage 2) considering serum albumin as a continuous variable

Variable	sHR (95% CI)	P
Age in years	1.03 (1.00-1.06)	0.08
Serum albumin in g/dl	0.57 (0.34-0.95)	0.03
9 MU colistimethate loading-dose	1.95 (0.95-3.99)	0.07

KDIGO, Kidney Disease: Improving Global Outcomes [1]; sHR, subdistribution hazard ratio; MU, million units.

Only results for variables retained in the final multivariable model are presented

Table S4. Univariable analysis of factors associated with acute kidney injury (KDIGO stage 3)

Variable	sHR (95% CI)	P
Age in years	1.00 (0.97-1.03)	0.95
Male gender	1.39 (0.38-5.18)	0.62
Diabetes mellitus	0.95 (0.26-3.5)	0.93
Chronic renal failure	1.17 (0.25-5.49)	0.85
Severe hepatic failure	2.5 (0.56-11.2)	0.23
Solid neoplasms	1.5 (0.40-5.62)	0.54
Hematological malignancies	No events	-
Causative agent of BSI		0.42
Enterobacteriaceae	1 (Reference)	
Pseudomonas spp.	0.69 (0.09-5.61)	
Acinetobacter spp.	1.02 (0.21-4.99)	
Enterobacteriaceae plus *Pseudomonas* spp.	2.98 (0.34-26.55)	
Enterobacteriaceae plus *Acinetobacter* spp.	5.01 (0.74-33.97)	
Polymicrobial	0.81 (0.18-3.73)	0.79
Colistin susceptibility test method		0.18
Vitek 2	1 (Reference)	
e-test	2.23 (0.69-7.23)	
ICU stay	0.59 (0.13-2.71)	0.5
Presence of central venous catheter	0.63 (0.17-2.33)	0.48
Presence of septic shock	0.75 (0.20-2.77)	0.66
Neutropenia	No events	-
Serum hemoglobin in g/dl	1.1 (0.77-1.58)	0.59
Serum total bilirubin in mg/dl	0.86 (0.67-1.11)	0.24
Serum creatinine in mg/dl	0.94 (0.29-2.98)	0.91
Serum albumin in g/dl	0.46 (0.20-1.05)	0.064
Serum albumin < 2.5 g/dl	3.16 (0.85-11.8)	0.086
Type of adequate therapy	1.06 (0.87-1.30)	0.87
Colistin monotherapy	1 (Reference)	

2 active agents including colistin	1.16 (0.34-3.97)	
3 active agents including colistin	1.73 (0.21-14.04)	
Time to adequate therapy in days	1.1 (0.91-1.33)	0.34
9 MU colistimethate loading-dose	2.46 (0.66-9.24)	0.18
Use of other nephrotoxic agents	0.84 (0.11-6.25)	0.86

KDIGO, Kidney Disease: Improving Global Outcomes [1]; sHR, subdistribution hazard ratio; CI, confidence intervals; BSI, bloodstream infection; ICU, intensive care unit; MU, million units.

Figure S1. Colistin binding to HSA evaluated by measuring the MG1655 *E. coli* strain growth.

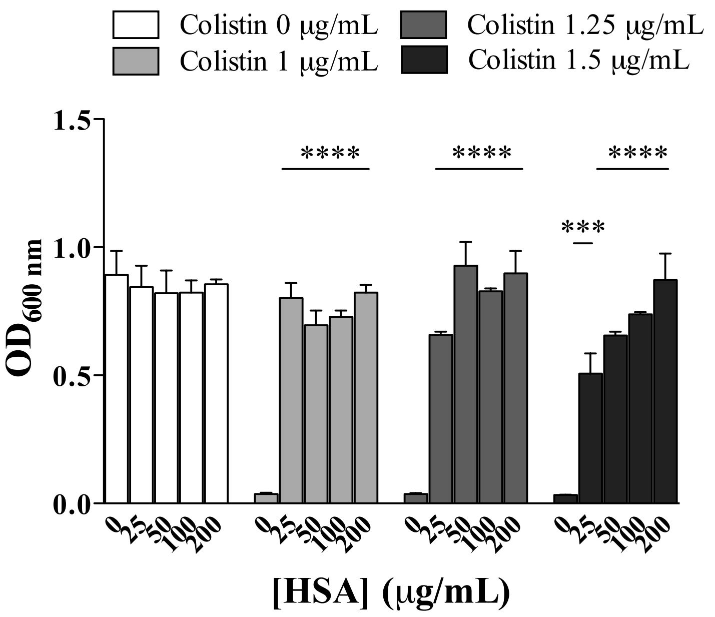

Figure S1 legend. HSA sequesters colistin from the *E.coli* growth medium, inhibiting its anti-microbial action. The activity of colistin (1, 1.25, and 1.5 μg/mL) on the MG1655 *E. coli* strain was tested in the absence or presence of increasing concentrations of HSA (25, 50, 100, and 200 μg/mL). Bacteria growth was measured at the wavelength of 600 nm. Data are presented as means ± SD (Student's t-test-, *** $p \leq 0.001$; **** $p \leq 0.0001$ compared to the relative control).

References

1. Kellum JA, Lameire N, Group KAGW. Diagnosis, evaluation, and management of acute kidney injury: a KDIGO summary (Part 1). Critical care. 2013; 17(1):204.
